# Supplementary material for: Claustrum and dorsal endopiriform cortex complex cell-identity is determined by Nurr1 and regulates hallucinogenic-like states in mice
Source: Nat Commun. 2024 Sep 18;15:8176. doi: 10.1038/s41467-024-52429-9 (PMC11408527; doi:10.1038/s41467-024-52429-9)
Supplement: Supplementary file 1 — Supplementary Information [file 41467_2024_52429_MOESM1_ESM.pdf]

Supplementary Information

Supplementary Figures

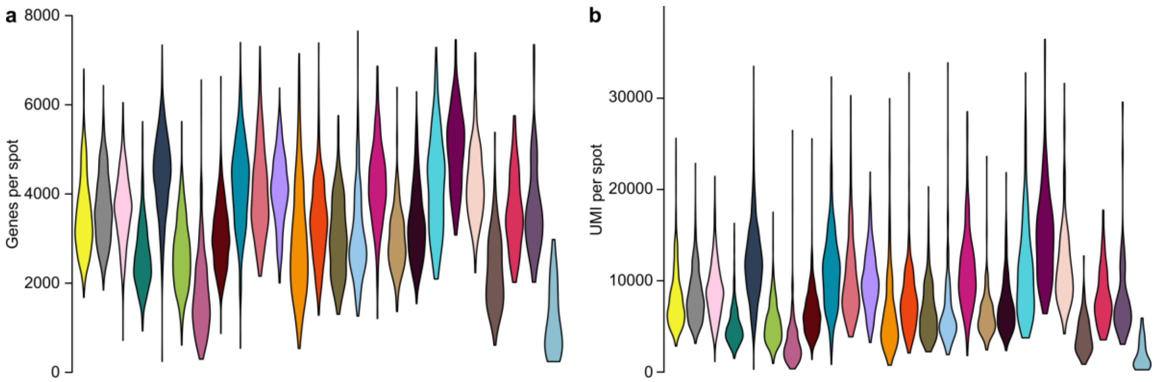

Supplementary Fig.1: Genes and UMI per spot per Cluster. (a, b) Violin plots showing the number of genes **(a)** and the UMI per spot **(b)** in each cluster. Abbreviations: UMI: unique molecular identifier.

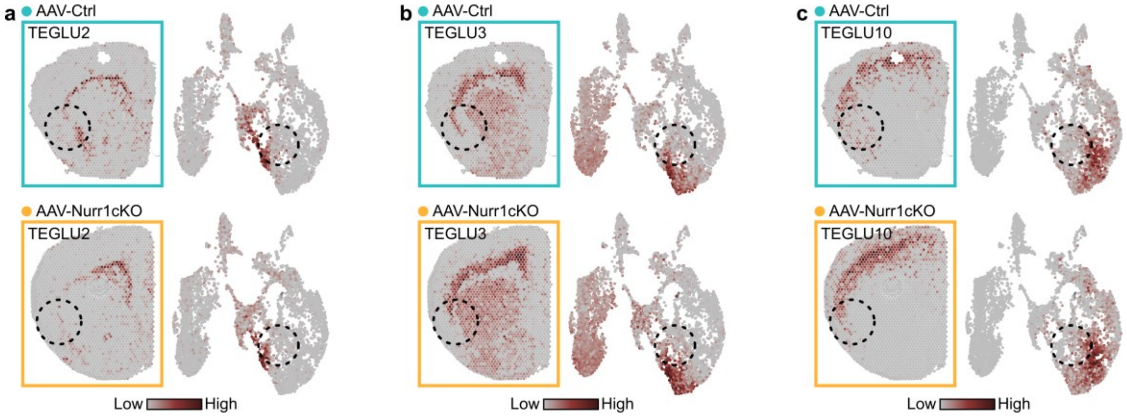

Supplementary Fig.2: Visualization of CLA-adjacent cortical layer specific clusters in AAV-Nurr1cKO mice. (a-c) On-tissue (left) and 2D-UMAP (right) visualization of TEGLU2 (L6b-specific) **(a)**, TEGLU3 (L6a-specific) **(b)** and TEGLU10 (L5-specific) **(c)** from AAV-Ctrl and AAV-Nurr1cKO sections. Abbreviations: AAV: adeno-associated virus, CLA: claustrum/dorsal endopiriform coprtex, TEGLU: telencephalon excitatory projecting neurons, L6b: critical layer 6b, L6a: cortical layer 6a, L5: cortical layer 5.

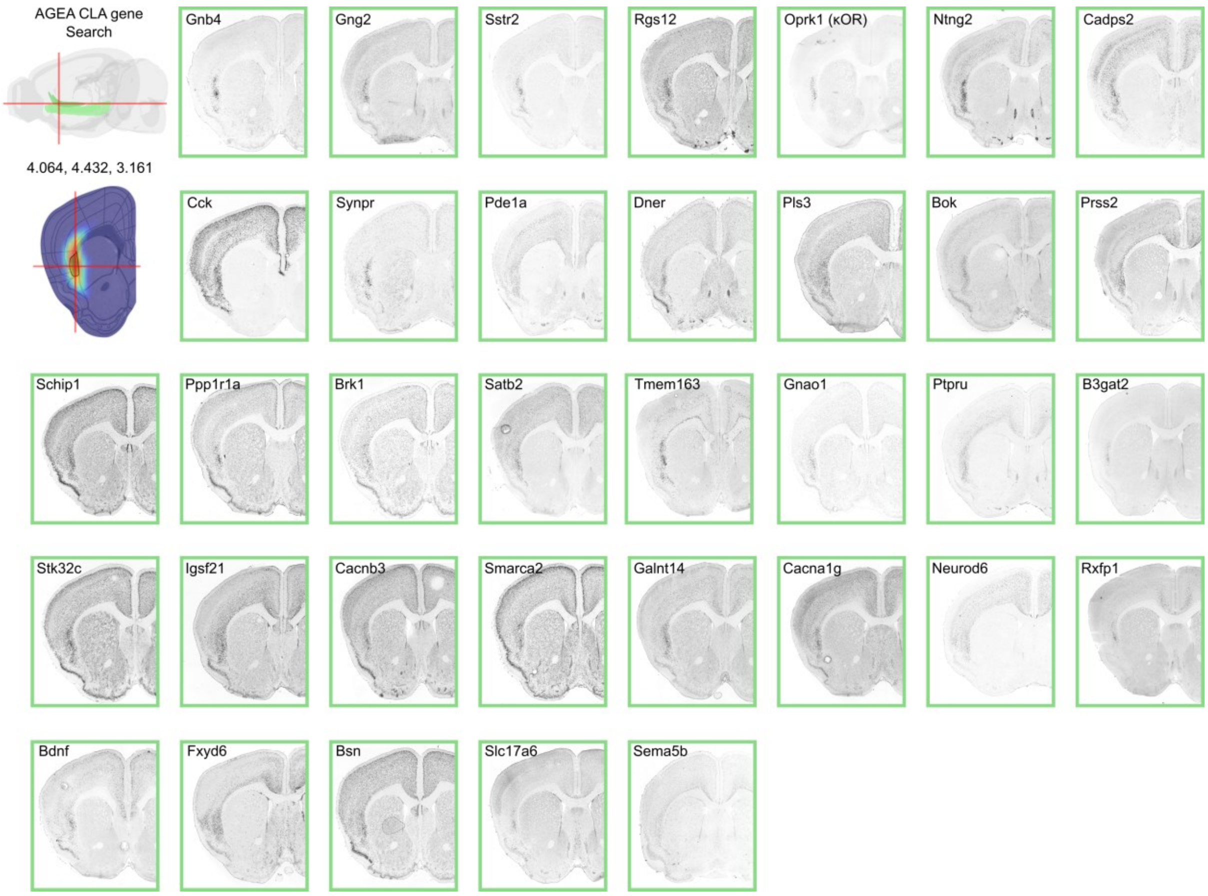

Supplementary Fig.3: Selected CLA enriched genes according to AGEA Fine Structure Search of the Allen brain atlas. Illustration showing the brain location that was used for searching of genes that are enriched in CLA region and representative ISH images of the CLA-specific genes that were selected from Allen brain atlas ISH database. Abbreviations: CLA: claustrum/dorsal endopiriform cortex complex

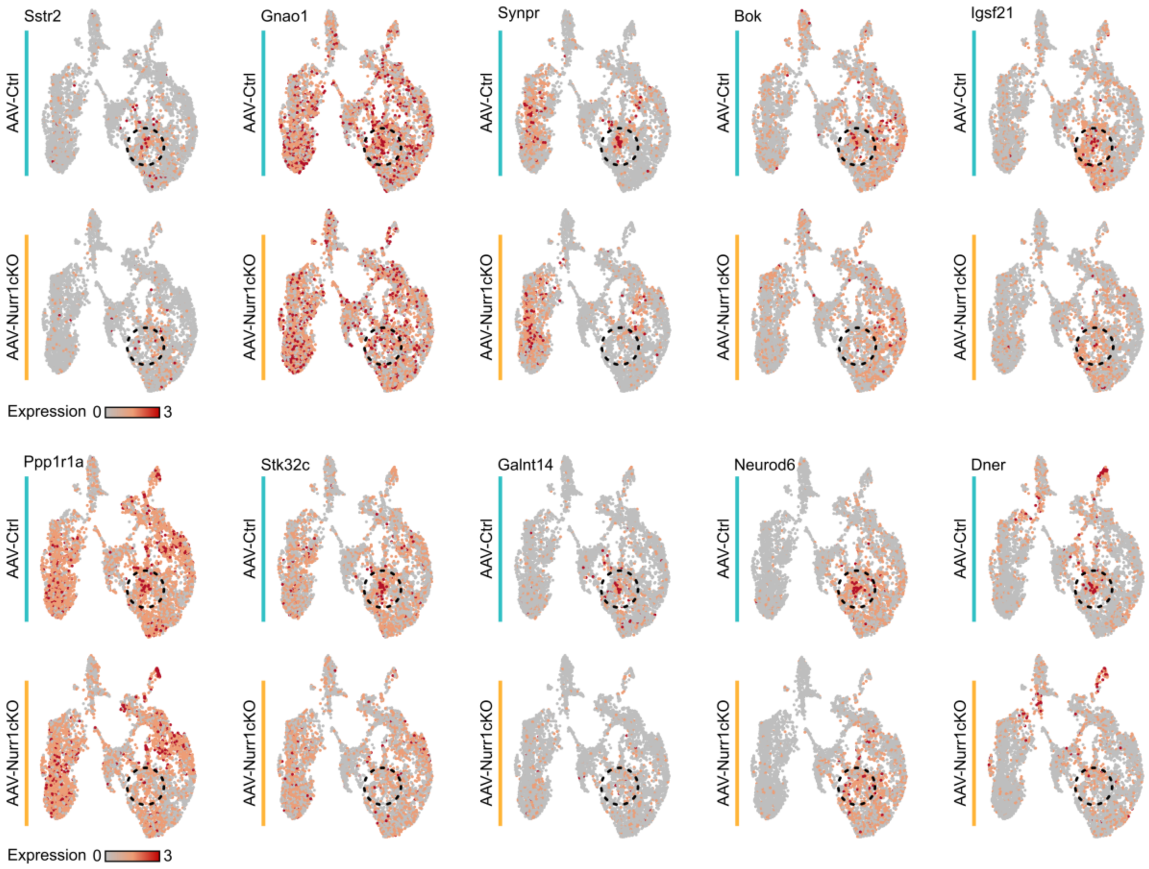

Supplementary Fig.4: 2D-UMAPs from CLA enriched genes. Visualization of the expression of several CLA enriched genes in the 2D-UMAP space from AAV-Ctrl and AAV-Nurr1cKO sections. The dashed circle denotes the location in 2D-UMAP of CLA spots from AAV-Ctrl mice. Abbreviations: AAV: adeno-associated virus.

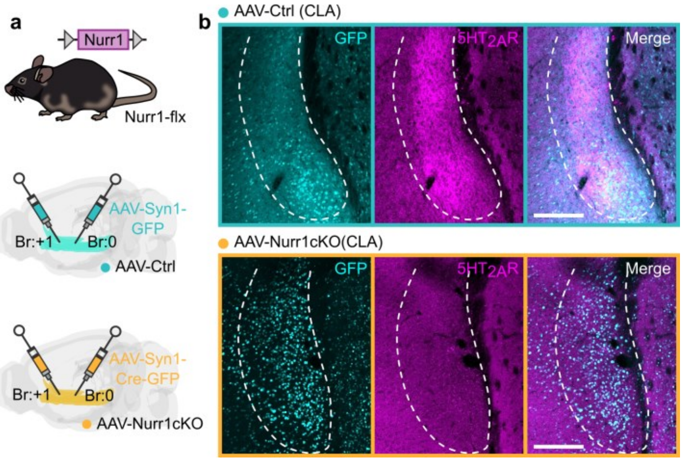

Supplementary Fig.5: AAV-Nurr1cKO mice display diminished $5HT_{2A}R$ protein levels in CLA. (a) Illustration of Nurr1-flx mouse line construct and the viral strategy of Nurr1 deletion in CLA. **(b)** Fluorescent images showing GFP and $5HT_{2A}R$ expression in CLA of AAV-Ctrl and AAV-Nurr1cKO mice (scale bar: 200 μm). Abbreviations: AAV: adeno-associated virus, CLA: claustrum/dorsal endopiriform coprtex, GFP: green fluorescent protein.

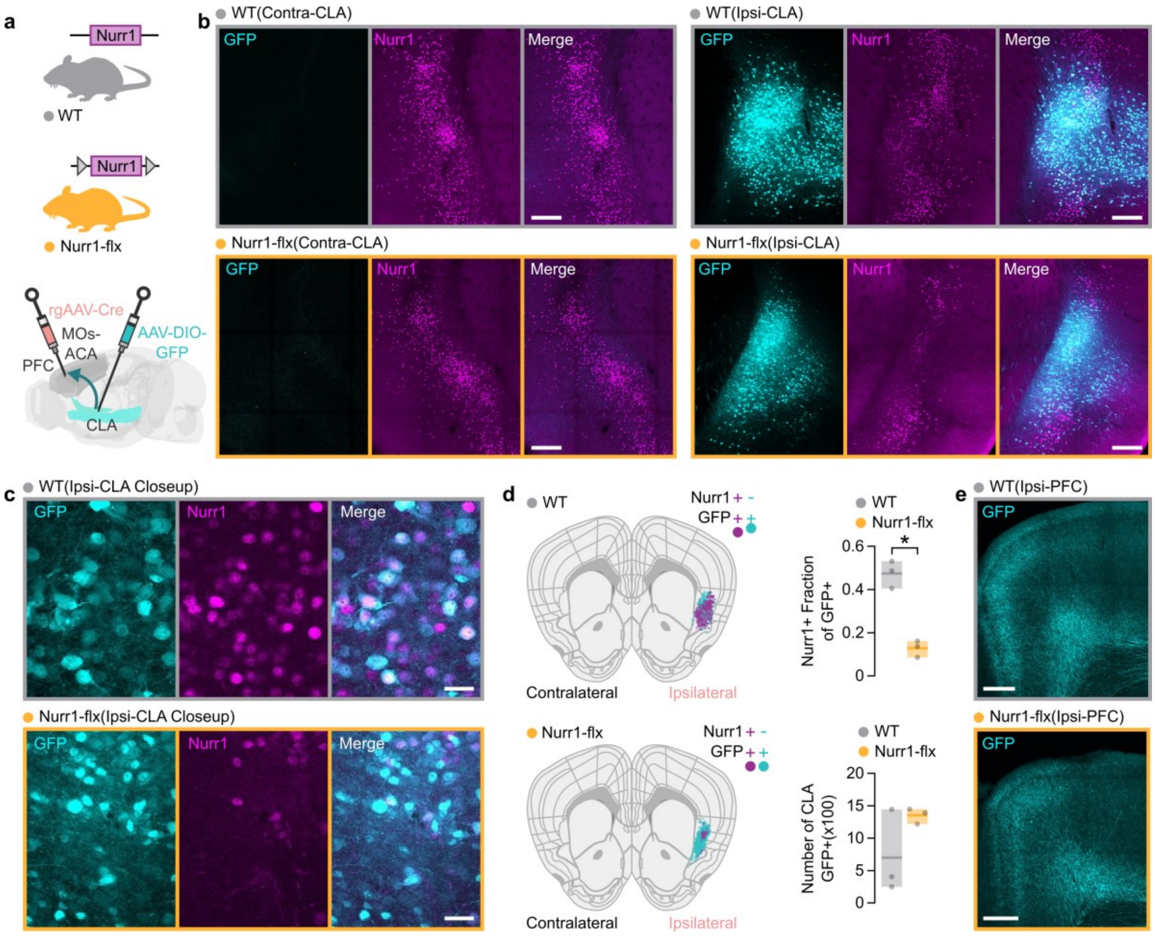

Supplementary Fig. 6. Retrograde deletion of Nurr1 and labelling of CLA-cortical prohecting cells in Nurr1-flx mice. (a) Illustration showing the WT and Nurr1-flx mice subjected to simultaneous injections of the rgAAV-Cre in PFC and the AAV-DIO-GFP in CLA. **(b)** Fluorescent images showing GFP and Nurr1 expression the ipsilateral and contralateral to the injections CLAs of WT and Nurr1-flx mice (scale bar: 200 μm). **(c)** Closeup fluorescent pictures showing GFP and Nurr1 expression in the ipsilateral to the injections CLAs of WT and Nurr1-flx mice (scale bar: 20 μm). **(d)** Illustration showing the localization of GFP positive and Nurr1/GFP double positive cells in the ipsilateral CLAs of WT and Nurr1-flx mice (left). Boxplots showing the fraction of Nurr1 positive cells in GFP labelled population and the absolute number (counted from 3 sections with 50 μm thickness) of GFP labelled population in the CLAs of WT and Nurr1-flx mice (WT: n=3, Nurr1-flx: n=3; *p=0.0013, Two-tailed Unpaired t-test. **(e)** Fluorescent images showing the GFP labelled fibers in PFC of WT and Nurr1-flx mice. Bar-graphs are expressed as mean ± minima/maxima. Abbreviations: rgAAV: retrograde adeno-associated virus. WT: wild type, MOs: secondary motor area, ACA: anterior cingulate area, PFC: prefrontal cortex, CLA: claustrum/dorsal endopiriform coprtex, GFP: green fluorescent protein. Source data are provided as a Source Data file.

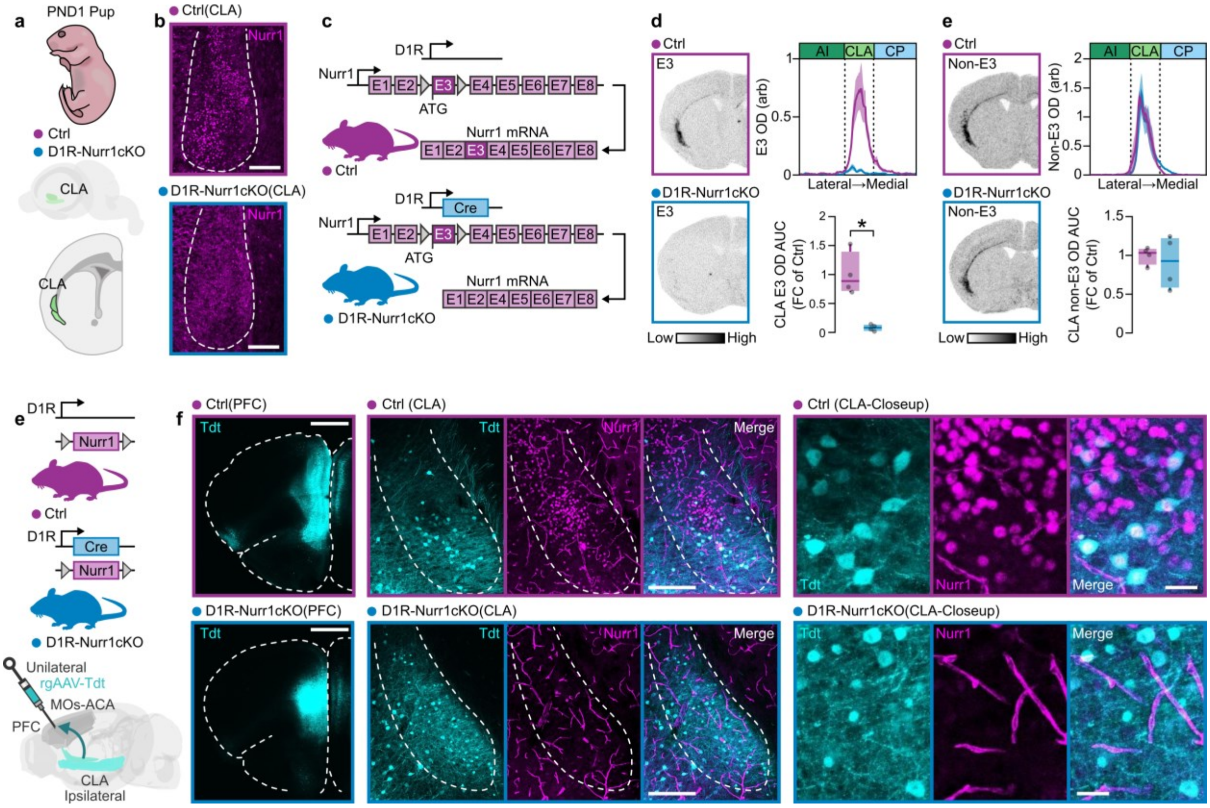

Supplementary Fig.7: PND1 CLA Nurr1 levels, non-coding Nurr1 mRNA expression and retrograde tracing of CLA cells in D1R-Nurr1cKO mice. (a) Fluorescent images showing Nurr1 immunolabelling in CLA of Ctrl and D1R-Nurr1cKO PND1 pups. **(b)** Illustration of the detailed D1R-Nurr1cKO mouse construct. **(c)** ISH autoradiographs (left) and graphs (right), showing the mRNA signal obtained from the E3 targeting Nurr1 probe at CLA of Ctrl and D1R-Nurr1cKO mice (Ctrl: n=4, D1R-Nurr1cKO: n=4; *p<0.0001, Two-tailed Unpaired t-test). **(d)** ISH autoradiographs (left) and graphs (right), showing the mRNA signal obtained from the Non-E3 targeting Nurr1 probe at CLA of Ctrl and D1R-Nurr1cKO mice (Ctrl: n=4, D1RNurr1cKO: n=4). **(e)** Illustration showing the experimental setup unilateral retrograde tracing of CLA with rgAAV-Tdt. **(f)** Fluorescent images showing the rgAAV-Tdt injection site in PFC (left), and the retrogradely labelled CLA cells (center: low magnification, right: high magnification) in Ctrl and D1R-Nurr1cKO mice. Nurr1 immunofluorescence showing double Nurr1/Tdt+ cells in Ctrl but not in D1R-Nurr1cKO mice (left scale bar: 1mm, central scale bar: 200µm, right scale bar: 20µm). Data from line-graphs are expressed as mean±SEM. Abbreviations: PND1: postnatal day 1 E: exon, ATG: starting codon, CLA: claustrum/dorsal endopiriform cortex complex, AI: agranular insula, CP: caudoputamen, OD: optic density, arb:

arbitrary units, AUC: area under the curve, FC: fold change. Source data are provided as a Source Data file.

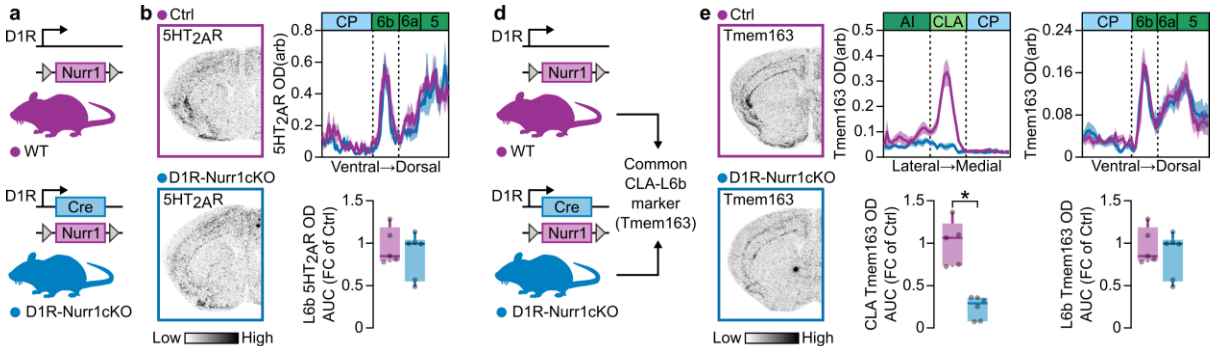

Supplementary Fig.8: 5HT$_{2A}$R and Tmem163 expression is unchanged in L6b of D1R-Nurr1cKO mice. (a) Illustration of D1R-Nurr1cKO mouse strain construct. (b) Left: ISH autoradiographs showing 5HT$_{2A}$R mRNA at CLA of Ctrl and D1R-Nurr1cKO mice (Ctrl: n=5, D1R-Nurr1cKO: n=6). (c) Illustration showing the selection of Tmem163 as a suitable marker to compare Nurr1 induced changes in CLA and L6b of D1R-Nurr1cKO mice. (d) Left: ISH autoradiographs showing the distribution of Tmem163 in Ctrl and D1R-Nurr1cKO mice. Center: Line-graph (upper) and boxplot (lower) showing the Tmem163 mRNA OD quantification in CLA (Ctrl: n=5, D1R-Nurr1cKO: n=6; *p=0.0002, Two-tailed Unpaired t-test). Right: Line-graph (upper) and boxplot (lower) showing the Tmem163 mRNA OD quantification in L6b (Ctrl: n=5, D1R-Nurr1cKO: n=6). Data from line-graphs are expressed as mean ± SEM. Boxplots show all data points, the 25th and 75th percentile (box), the median (center) and the maxima (whiskers). Abbreviations: L6b: cortical layer 6b, CLA: claustrum/dorsal endopiriform cortex complex, AI: agranular insula, CP: caudoputamen, OD: optic density, arb: arbitrary units, AUC: area under the curve, FC: fold change. Source data are provided as a Source Data file.

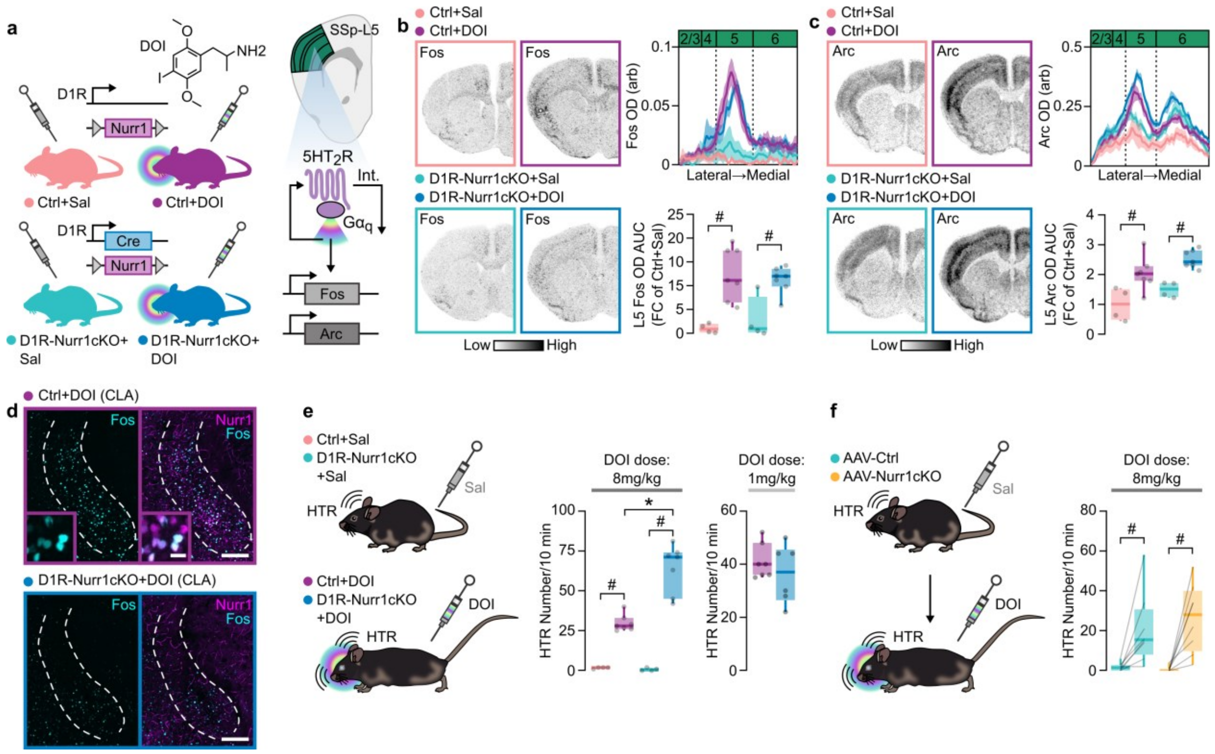

Supplementary Fig.9: DOI response after Nurr1 deletion in CLA. (a) Left: Illustration of the systemic DOI (8 mg/kg ip) administration experimental set up. Right: Illustration of $5HT_2R$-mediated effects on the SSp-L5. **(b)** ISH autoradiographs (left) and graphs (right) showing Fos mRNA in SSp-L5 of Ctrl and D1R-Nurr1cKO mice treated with DOI. (Ctrl+Sal: n=4, Ctrl+DOI: n=7, D1R Nurr1cKO+Sal: n=4, D1RNurr1cKO+DOI: n=7; two-way ANOVA, Treatment: $F(1,18)=30.15$, $p<0.0001$; Sal vs DOI: #$p<0.01$, Sidak's post-hoc test). **(c)** ISH autoradiographs (left) and graphs (right) showing Arc mRNA in SSp-L5 of Ctrl and D1R-Nurr1cKO mice treated with DOI. (Ctrl+Sal: n=4, Ctrl+DOI: n=7, D1R-Nurr1cKO+Sal: n=4, D1R-Nurr1cKO+DOI: n=7; two-way ANOVA, Treatment: $F(1,18)=28.12$, $p<0.0001$; Sal vs DOI: #$p<0.05$, Sidak's posthoc test). **(d)** Immunofluorescence in CLA showing double Fos/Nurr1 double positive cells in Ctrl mice but not in D1R-Nurr1cKO mice treated with DOI (scale bar: 100µm, inlet scale bar: 20µm). **(e)** Illustration (left) showing the experimental setup of DOI-induced (8 mg/kg or 1 mg/kg) HTR and boxplot (right) showing the 10 min HTR count of Ctrl and D1R-Nurr1cKO (Ctrl+Sal: n=4, Ctrl+DOI: n=7, D1R-Nurr1cKO+Sal: n=4, D1RNurr1cKO+DOI: n=7; two-way ANOVA, Genotype×Treatment: $F(1,18)=18.83$, $p=0.0004$; Ctrl vs D1R-Nurr1cKO: *$p<0.0001$, Sal vs DOI: #$p<0.001$, Sidak's post-hoc test). **(f)** Illustration (left) showing the experimental setup of DOI-induced (8 mg/kg) HTR and boxplot (right) showing the 10 min HTR count of AAV-Ctrl and D1R-Nurr1cKO mice (AAV-Ctrl: n=8, AAV-Nurr1cKO: n=8; repeated measures two-way ANOVA, Treatment: $F(1,14)=27.19$, $p=0.0001$; Sal vs DOI: #$p<0.01$, Sidak's post-hoc test). Data in line-graphs are

expressed as mean ± SEM. Boxplots show all data points, the 25th and 75th percentile (box), the median (center) and the maxima (whiskers). Abbreviations: DOI: 2,5-dimethoxy-4-iodoamphetamine, ip: intraperitoneal, SSp: primary somatosensory area, Sal: saline, CLA: claustrum/dorsal endopiriform cortex complex, arb: arbitrary units, AUC: area under the curve, FC: fold change, HTR: head twitch response, AAV: adenoassociated virus. Source data are provided as a Source Data file.

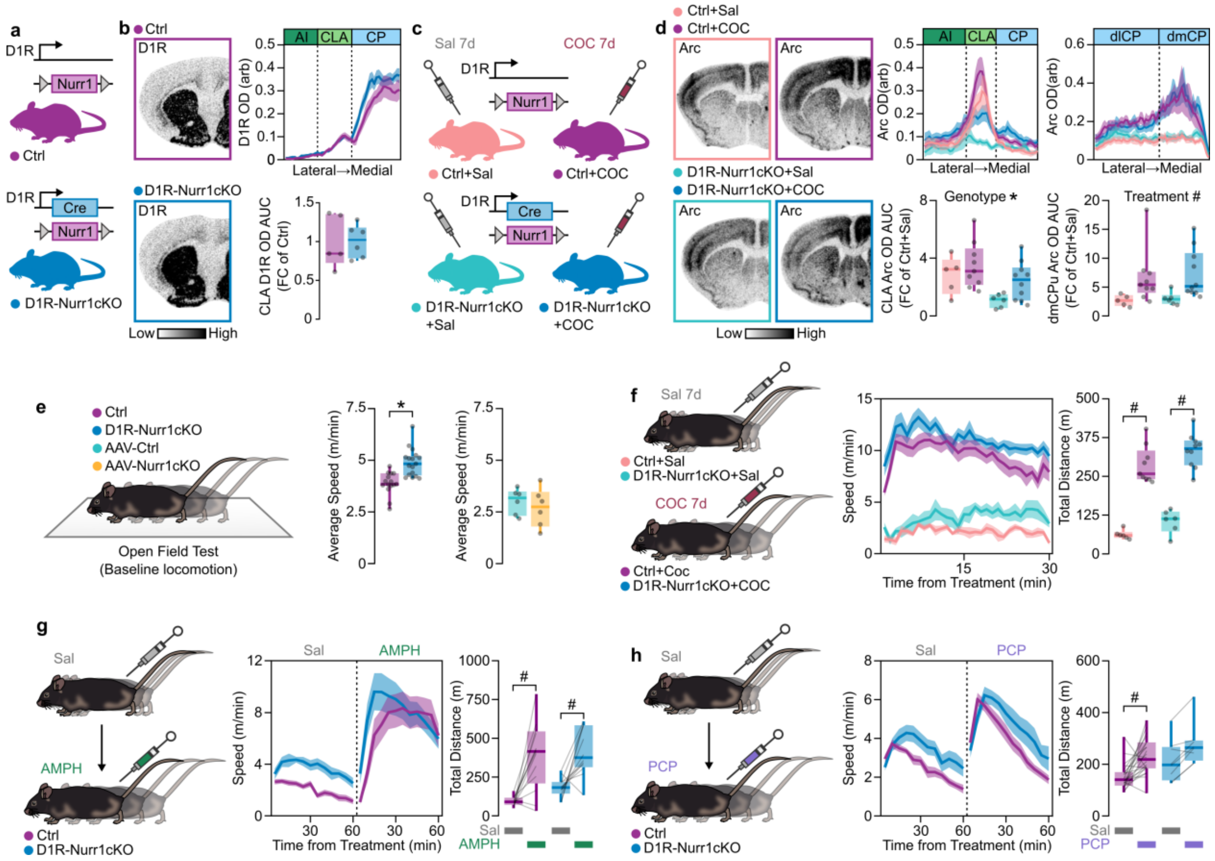

Supplementary Fig.10. Psychostimulant effects on D1R-Nurr1cKO mice. (a) Schematic depiction of the D1-Nurr1cKO mouse line. **(b)** ISH Autoradiographs and graphs showing the expression of D1R in the CLA of Ctrl and D1-Nurr1cKO mice (Ctrl: n=5, D1R-Nurr1cKO: n=6). **(c)** Illustration of the subchronic COC (10mg/kg) administration. (d) ISH autoradiographs and graphs showing the expression of Arc expression in CLA (left) and dmCP (right) of Ctrl and D1-Nurr1cKO mice after the subchronic administration of cocaine (Ctrl+Sal: n=6, Ctrl+COC: n=9, D1R-Nurr1cKO+Sal: n=6, D1R-Nurr1cKO+DOI: n=11; CLA: Two-way ANOVA, Treatment: $F(1, 26)=8.099$, *p=0.0085; dmCP: Two-way ANOVA, Treatment: $F(1, 26)=9.022$, #p=0.0058). **(e)** Schematic depiction of OFT (left) and boxplots (right) showing the baseline average locomotion speed of Ctrl, D1-Nurr1cKO, AAV-Ctrl and AAV-Nurr1cKO mice (Ctrl: n=15, D1R-Nurr1cKO: n=17; *p<0.0001, Two-tailed Unpaired t-test). **(f)** Schematic depiction of OFT after subchronic Sal or COC treatment (left). Graphs showing the Speed (center) and the total distance covered (right) of Ctrl and D1-Nurr1cKO mice treated with Sal or COC for 7 days (Ctrl+Sal: n=6, Ctrl+COC: n=9, D1R-Nurr1cKO+Sal: n=6, D1R-Nurr1cKO+DOI: n=11; Two-way ANOVA, Treatment: $F(1, 27)=148.5$, $p<0.0001$, Genotype: $F(1, 27)=6.086$, $p<0.05$; Sal vs COC: #p<0.0001, Sidak's post-hoc test). **(g)** Schematic depiction of OFT after acute Sal and AMPH treatment (left). Graphs showing the Speed

(center) and the total distance covered (right) of Ctrl and D1-Nurr1cKO after the acute treatment of Sal and AMPH (Ctrl: n=13, D1R-Nurr1cKO: n=12; Repeated measures Two-way ANOVA, Treatment: $F(1, 23)=39.36$, $p<0.0001$; Sal vs AMPH: #$p<0.01$, Sidak's post-hoc test). Schematic depiction of OFT after acute Sal and PCP treatment (left). Graphs showing the Speed (center) and the total path-distance (right) of Ctrl and D1-Nurr1cKO after Sal and PCP (Ctrl: n=22, D1R-Nurr1cKO: n=8; Repeated measures Two-way ANOVA, Treatment: $F(1, 28)=18.27$, $p<0.001$; Sal vs PCP: #$p<0.001$, Sidak's post-hoc test). Line-graphs' data are expressed as mean±SEM. Boxplots show all data points, the 25th and 75th percentile (box), the median (center) and the maxima (whiskers). Abbreviations: ISH: in situ hybridization, CLA: claustrum/dorsal endopiriform cortex complex, AI: agranular insula, CP: caudoputamen, dlCP: dorsolateral caudoputamen, dmCP: dorsomedial caudoputamen, Sal: saline, COC: cocaine, 7d: 7 days, OFT: open field test, AMPH: amphetamine, PCP: phencyclidine. Source data are provided as a Source Data file.

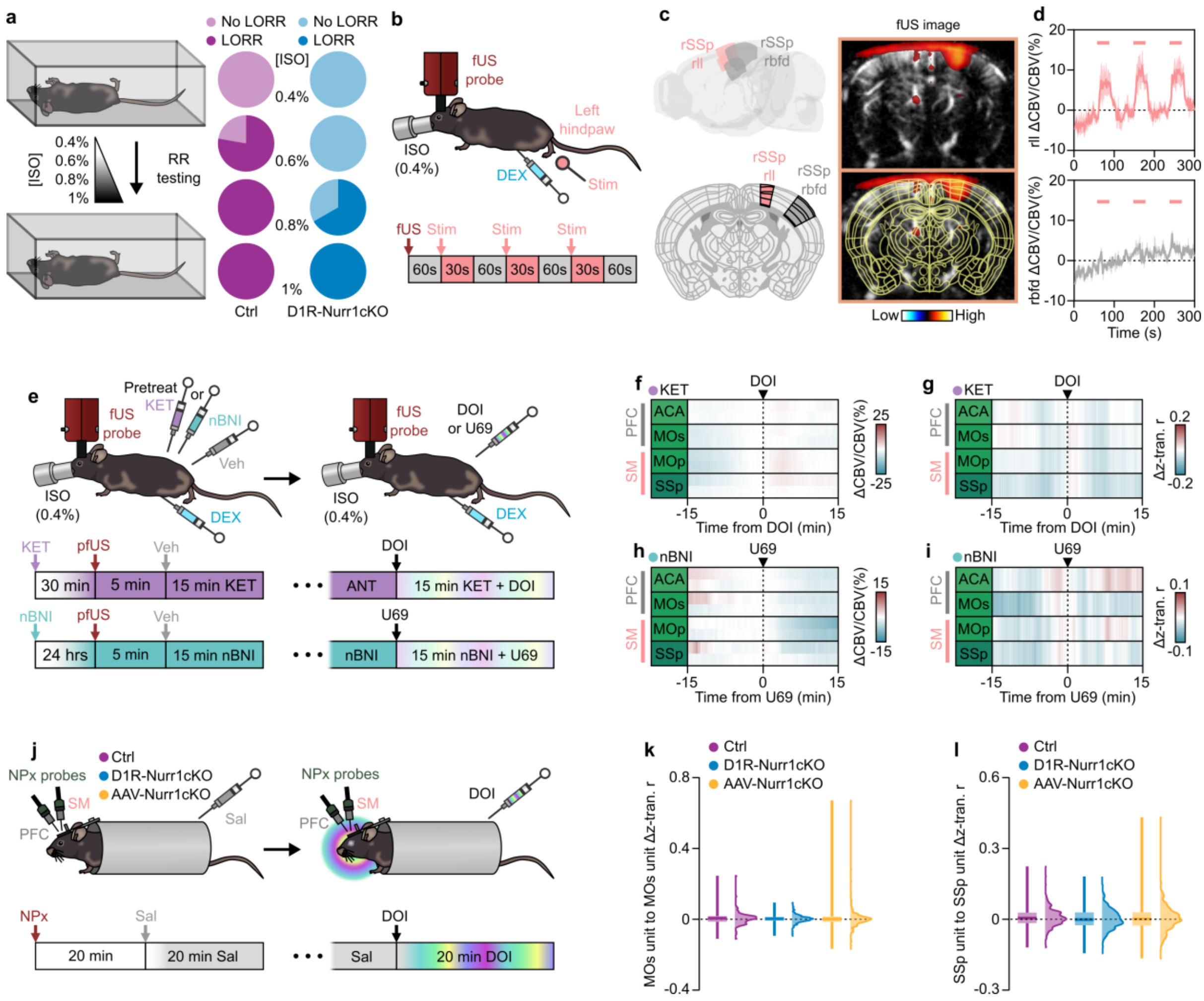

Supplementary Fig.11: Validation of the pfUS sedation protocol, the effects of 5HT2R or κOR antagonists on pfUS, and within-region unit correlation in neuropixel recordings. **(a)** Left: Illustration showing the the ISO sensitivity experiment. Right: Pie-charts showing the percentage of Ctrl and D1R-Nurr1cKO mice that exhibited LORR at each ISO concentration. **(b)** Illustration showing the fUS recording and the tactile stimulus protocol. **(c)** Left: Schematic depiction of selected brain regions (rSSp-ll: pink, rSSp-bfd: gray). Right: fUS images showing the activation of R SSp-ll after the tactile-stimulus. **(d)** Line-graphs (n=3) showing CBV changes in rSSp-ll and rSSp-bfd after the left-hindpaw stimulation. **(e)** Illustration showing the pfUS recordings with pretreatment of KET (1mg/kg ip) or nBNI (32 mg/kg ip) and the treatment with DOI (1 mg/kg ip) or U69 (1 mg/kg sc) respectively. **(f)** Heatmap showing CBV changes over time after KET and DOI combination (n=7). **(g)** Heatmap showing the the mean Δz-transformed r-coefficient (correlation with the rest cortical regions) change over time after KET and DOI combination (n=7). **(h)** Heatmap showing CBV changes over time after nBNI and U69 combination (n=8). **(i)** Heatmap showing the mean Δz-transformed r-coefficient (correlation with the rest cortical regions) change over time after nBNI and U69 combination

(n=8). **(j)** Illustration showing the neuropixel-electrophysiological recordings. **(k, l)** Boxplot/violin plot graph showing the within-region unit Δz-transformed r-coefficient in MOs **(k)** and SSp **(l)** of Ctrl, D1-Nurr1cKO and AAV-Ctrl mice (Ctrl mice: n=3, Ctrl units: n=281, D1R-Nurr1cKO mice: n=4, D1R-Nurr1cKO units: n=387, AAV-Nurr1cKO mice: n=3, AAV-Nurr1cKO units: n=351. Data in line-graphs are expressed as mean ± SEM. Boxplots show all data points, the 25th and 75th percentile (box), the median (center) and the maxima (whiskers). Abbreviations: ISO: isoflurane, RR: righting reflex, LORR: loss of righting reflex, pfUS: pharmacological functional ultrasound, DEX: dexmedetomidine, Stim: tactile stimulus, ll: lower limb, rSSp: right primary somatosensory area, bfd: barrel field, KET: ketanserin, nBNI: nor-binaltorphimine, Veh: vehicle, DOI: 2,5-dimethoxy-4-iodoamphetamine, ip: intraperitoneal, sc: subcutaneous, SM: sonsorimotor, PFC: prefrontal cotex, NPx: neuropixel, Sal: saline, MOs: secondary motor area. Source data are provided as a Source Data file.
