# Supplementary material for: Claustrum and dorsal endopiriform cortex complex cell-identity is determined by Nurr1 and regulates hallucinogenic-like states in mice
Source: Nat Commun. 2024 Sep 18;15:8176. doi: 10.1038/s41467-024-52429-9 (PMC11408527; doi:10.1038/s41467-024-52429-9)
Supplement: Supplementary file 3 — Description of Additional Supplementary Files [file 41467_2024_52429_MOESM3_ESM.pdf]

Description of Additional Supplementary Files

Supplementary Data 1. Spots count per cluster. The table displays the number of spots for each cluster across three categories: within each cluster, across its respective samples, and across both sample types.

Supplementary Data 2. Marker genes for each cluster from the Nurr1 dataset clustering analysis. These genes were used for clusters identification. Multiple-testing adjusted P-values computed by PRESTO.

Supplementary Data 3. Differentially expressed genes in AAV-Nurr1cKO compared to the AAV-Ctrl for the CLA selected spots. Multiple-testing adjusted P-values computed by PRESTO.
