# Supplementary material for: Claustrum and dorsal endopiriform cortex complex cell-identity is determined by Nurr1 and regulates hallucinogenic-like states in mice
Source: Nat Commun. 2024 Sep 18;15:8176. doi: 10.1038/s41467-024-52429-9 (PMC11408527; doi:10.1038/s41467-024-52429-9)
Supplement: Supplementary file 7 — Reporting Summary [file 41467_2024_52429_MOESM7_ESM.pdf]

nature portfolio

Corresponding author(s): Ioannis Mantas

Last updated by author(s): Aug 10, 2024

Reporting Summary

Nature Portfolio wishes to improve the reproducibility of the work that we publish. This form provides structure for consistency and transparency in reporting. For further information on Nature Portfolio policies, see our Editorial Policies and the Editorial Policy Checklist.

Statistics

For all statistical analyses, confirm that the following items are present in the figure legend, table legend, main text, or Methods section.

n/a	Confirmed	
☐	☒	The exact sample size (*n*) for each experimental group/condition, given as a discrete number and unit of measurement
☐	☒	A statement on whether measurements were taken from distinct samples or whether the same sample was measured repeatedly
☐	☒	The statistical test(s) used AND whether they are one- or two-sided<br>*Only common tests should be described solely by name; describe more complex techniques in the Methods section.*
☒	☐	A description of all covariates tested
☐	☒	A description of any assumptions or corrections, such as tests of normality and adjustment for multiple comparisons
☐	☒	A full description of the statistical parameters including central tendency (e.g. means) or other basic estimates (e.g. regression coefficient) AND variation (e.g. standard deviation) or associated estimates of uncertainty (e.g. confidence intervals)
☐	☒	For null hypothesis testing, the test statistic (e.g. *F*, *t*, *r*) with confidence intervals, effect sizes, degrees of freedom and *P* value noted<br>*Give P values as exact values whenever suitable.*
☒	☐	For Bayesian analysis, information on the choice of priors and Markov chain Monte Carlo settings
☒	☐	For hierarchical and complex designs, identification of the appropriate level for tests and full reporting of outcomes
☒	☐	Estimates of effect sizes (e.g. Cohen's *d*, Pearson's *r*), indicating how they were calculated

Our web collection on statistics for biologists contains articles on many of the points above.

Software and code

Policy information about availability of computer code

Data collection	Spatial Transcriptomics data collection: Fastq files were generated from the experiments using instrument model Illumina Novaseq 2000. The processed data was generated using custom script which, for reproducibility, can be found in the github repository: https://github.com/giacomellolab/CLA-Nurr1.<br>Animal Behavior data collection: EthoVision XT 8 (Noldus)<br>Functional Ultrasound data collection: Icoscan (Iconeus)<br>Slice electrophysiology data collection: pClamp 10 software (Axon Instruments)<br>Neuropixel probe electrophysiology data collection: SpikeGLX (Bill Karsh, Janelia)
Data analysis	In situ Hybridization and Immunofluorescent image analysis: Fiji (National Institute of Health)<br>Graph Visualization and Statistics: GraphPad Prism 7.0 (GraphPad Software Inc.)<br>Functional ultrasound analysis: Matlab (2019a)<br>Spatial Transcriptomic analysis: The counts matrices from the sample fastq sequences were generated using Spaceranger count (v1.3.1, 10x Genomics Inc.). The generated counts matrices were then processed and analyzed using the R package Seurat (v4.1.1), Harmony (v0.1.0) and SCTransform (v0.3.4). Visium spots falling under the CLA region were manually selected in Loupe Browser (v6.3.0, 10x Genomics Inc.) using prior knowledge of the mouse brain tissue anatomy. Differential expression (DE) analysis was then performed for these manually picked spots between the AAV-Nurr1cKO sections and the AAV-Ctrl sections.<br>Bulk RNA sequencing analysis: The reference transcriptome Mus_musculus.GRCm39.cdna.all (release-107) from Ensembl was used. Salmon/1.6.0 was used to create an index on the transcriptome which is used as a structure for quasi-mapping of RNA-seq reads during alignment and quantification (Avi Srivastava, Hirak Sarkar, Nitish Gupta, Rob Patro (2016) https://doi.org/10.1093/bioinformatics/btw277).

Salmon Index was made with parameters such as the --gcBias flag to correct for the common systematic biases in RNA-seq data, making it possible correct for fragment-level GC biases in the data. The other parameter was the --validateMappings flag for selective alignment.Next, “tximport” package version 1.20.0, was used to import and summarize the transcript abundances and construct of a transcript-level DESeqDataSet object from Salmon output files. This package was also used to construct a tx2gene table for linking transcripts to genes using Gencode Mouse Transcript Annotation (GTF file) Release M30 (GRCm39). Next, the necessary quantification data for DESeq2 was imported using the tximport function.
Finally, DESeq2 package version 1.34.0 was used to construct a DESeqDataSet from the txi object, using sample information and design = ~ condition (Love M, Huber W, Anders S Genome Biology (2014)).
Differential gene expression analysis (Ctrl vs D1R-Nurr1cKO) was done with results() function in the DESeq2 package.
'rtracklayer' package version 1.52.1 was used to extract “gene_id", "gene_name", and "transcript_id” from the Gencode annotation GTF file to later match them to the (DE) results gene IDs.
lfcShrink() function in DESeq2 package was used to estimate the log fold changes (LFCs) and shrink them toward zero to avoid the small counts values’ otherwise inaccurately estimated large LFC, from affecting and dominating the real high-ranked LFCs. The adaptive t prior shrinkage estimator type=“apeglm” was used from the apeglm package.
The 'EnhancedVolcano' package version 1.10.0 and the 'pheatmap' package version 1.0.12 were used to make volcano plots and the heatmap.
Neuropixel probe electrophysiological recordings: Spike sorting was performed using Kilosort 2.0 (Matlab 2018b). Probe tract reconstruction was performed using the DMC-BrainMap (https://github.com/hejDMC/napari-dmc-brainmap) napari plugin (napari version: 0.4.19; Python version: 3.8.19). Functional connectivity analysis was performed in Python (3.8.19) using the following packages: numpy (1.22.4), pandas (1.5.0), scipy (1.10.1), matplotlib (3.7.5), seaborn (0.11.0), spykes (0.3.dev0).

For manuscripts utilizing custom algorithms or software that are central to the research but not yet described in published literature, software must be made available to editors and reviewers. We strongly encourage code deposition in a community repository (e.g. GitHub). See the Nature Portfolio guidelines for submitting code & software for further information.

Data

Policy information about availability of data

All manuscripts must include a data availability statement. This statement should provide the following information, where applicable:

- Accession codes, unique identifiers, or web links for publicly available datasets
- A description of any restrictions on data availability
- For clinical datasets or third party data, please ensure that the statement adheres to our policy

Spatial Transcriptomics data: The necessary scripts to reproduce the Spatial Transcriptomics data analysis performed in this study can be found in the github repository: https://github.com/giacomellolab/CLA-Nurr1 which is publicly available. The sequencing data generated in this study have been deposited in NCBI-GEO database under accession code GSE229770 [https://www.ncbi.nlm.nih.gov/geo/query/acc.cgi?acc=GSE229770]. The corresponding count matrices, histology images, spotfiles from the CLA region, and final Seurat object from the ST data analysis are publicly available on Mendeley Data project (https://doi.org/10.17632/wxcbdvrh2t.1)75. The GEO accession number for the bulk RNA-seq is GSE229732.

Research involving human participants, their data, or biological material

Policy information about studies with human participants or human data. See also policy information about sex, gender (identity/presentation), and sexual orientation and race, ethnicity and racism.

Reporting on sex and gender	The human brain section that was used for radioactive ISH was acquired from University College of London, Queens Square Brain Bank. The current fresh frozen brain section was obtained from an 88 year old male (p31/11).
Reporting on race, ethnicity, or other socially relevant groupings	N/A
Population characteristics	N/A
Recruitment	N/A
Ethics oversight	The current human brain sample used in this study was obtained following informed consent from the donor or the donor's relatives. The study was approved by the appropriate ethical review board, with the ethical approval number 2014/1366-31.

Note that full information on the approval of the study protocol must also be provided in the manuscript.

Field-specific reporting

Please select the one below that is the best fit for your research. If you are not sure, read the appropriate sections before making your selection.

[x] Life sciences   [ ] Behavioural & social sciences   [ ] Ecological, evolutionary & environmental sciences

For a reference copy of the document with all sections, see nature.com/documents/nr-reporting-summary-flat.pdf

Life sciences study design

All studies must disclose on these points even when the disclosure is negative.

Sample size	In this study, we did not perform formal statistical calculations to predetermine sample sizes. Instead, our sample sizes were chosen based on established practices in the field and the nature of the experiments conducted. The selected sample sizes are consistent with previous studies of similar scope and complexity, where they have been sufficient to observe robust and reproducible effects. Additionally, these sample sizes were determined to balance practical considerations such as resource availability and experimental feasibility, while still providing meaningful and reliable data.
Data exclusions	In bulk RNA-seq, two sample with low mRNA counts were excluded from the analysis. In functional ultrasound experiments, mice with abnormal recording values and low quality ultrasound images were excluded from the study.
Replication	The number of independent biological replicates in each data panel is indicated in the Figure legends.
Randomization	Mice were assigned randomly into experimental groups and processed in an arbitrary order.
Blinding	All mice and subsequent samples were labelled only with mouse ID numbers and did not indicate genotype or type of treatment. Genotype or treatment type were decoded after the data acquisition and quantification analysis were complete. For all experiments, the investigators were blinded to group allocation during data collection and/or analysis

Reporting for specific materials, systems and methods

We require information from authors about some types of materials, experimental systems and methods used in many studies. Here, indicate whether each material, system or method listed is relevant to your study. If you are not sure if a list item applies to your research, read the appropriate section before selecting a response.

Materials & experimental systems

n/a	Involved in the study
☐	☒ Antibodies
☒	☐ Eukaryotic cell lines
☒	☐ Palaeontology and archaeology
☐	☒ Animals and other organisms
☒	☐ Clinical data
☒	☐ Dual use research of concern
☒	☐ Plants

Methods

n/a	Involved in the study
☒	☐ ChIP-seq
☒	☐ Flow cytometry
☒	☐ MRI-based neuroimaging

Antibodies

Antibodies used	Primary antibodies:<br>Rabbit anti-Nurr1 (Santa Cruz; Cat. No.: sc-990 (E-20)); dilution: 1:50<br>Rat anti-Fos (Synaptic Systems; Cat. No.: 226 017); dilution: 1:200<br>Mouse anti-Cre (MilliporeSigma; Cat. No.: MAB3120); dilution: 1:500<br>Rabbit anti-5HT2AR (Neuromics; Cat. No.:RA24288); dilution: 1:200
Validation	Antibodies were pre-established published commercial antibodies. See the following: https://www.neuromics.com/RA24288, https://doi.org/10.1038/npp.2010.195, https://www.sigmaaldrich.com/SE/en/product/mm/mab3120, https://www.sysy.com/product/226017, https://doi.org/10.1111/j.1471-4159.2006.03829.x, https://www.scbt.com/p/nurr1-nur77-antibody-e-20

Animals and other research organisms

Policy information about studies involving animals; ARRIVE guidelines recommended for reporting animal research, and Sex and Gender in Research

Laboratory animals	Sprague-Dawley rats (2-6 months) and mice with C57BL6 genetic background (2-6 months) were housed in air-conditioned rooms (12-h dark/light cycle) at 20°C and a humidity of 53%, with free access to food and water, provided ad libitum. Nurr1 heterozygous (+/!) mice were generated as previously described in Rojas et al., 2007. Our Nurr1-flx mouse line was generated by insertion of two loxP sequences in the second and third introns, so that the coding sequence of the first coding exon is excised at the presence of Cre. This mouse line was crossed with mice expressing Cre under D1R promoter. As conditional KO mice for each Cre-line were considered those which were heterozygous for Cre (+/-) and homozygous for Nurr1-floxed (+/+). The transgenic D1RCre (EY262) mouse line has been obtained from GENSAT. For the evaluation of Cre expression, D1RCre mice were crossed with a floxed mCherry mouse line and the combined heterozygous D1RCre (+/-) and floxed mCherry (+/-) offsprings were used for imaging.
Wild animals	None

Reporting on sex | Both male and female mice were used for the studies.

Field-collected samples | None

Ethics oversight | Housing and experimental procedures were fully compliant with the principles of Care and Use of Laboratory Animals.

Note that full information on the approval of the study protocol must also be provided in the manuscript.

Plants

Seed stocks | *Report on the source of all seed stocks or other plant material used. If applicable, state the seed stock centre and catalogue number. If plant specimens were collected from the field, describe the collection location, date and sampling procedures.*

Novel plant genotypes | *Describe the methods by which all novel plant genotypes were produced. This includes those generated by transgenic approaches, gene editing, chemical/radiation-based mutagenesis and hybridization. For transgenic lines, describe the transformation method, the number of independent lines analyzed and the generation upon which experiments were performed. For gene-edited lines, describe the editor used, the endogenous sequence targeted for editing, the targeting guide RNA sequence (if applicable) and how the editor was applied.*

Authentication | *Describe any authentication procedures for each seed stock used or novel genotype generated. Describe any experiments used to assess the effect of a mutation and, where applicable, how potential secondary effects (e.g. second site T-DNA insertions, mosiacism, off-target gene editing) were examined.*
